# Interpretation for scales of measurement linking with abstract algebra

**DOI:** 10.1186/2043-9113-4-9

**Published:** 2014-06-10

**Authors:** Jitsuki Sawamura, Shigeru Morishita, Jun Ishigooka

**Affiliations:** 1 Department of Psychiatry, Tokyo Women’s Medical University, Tokyo, Japan; 2 Depression Prevention Medical Center, Inariyama Takeda Hospital, Kyoto, Japan

**Keywords:** Scales on measurement, Stevens classification, Interpretation, Abstract algebra, Data-mining, Hierarchical cluster, Clinical medicine

## Abstract

The Stevens classification of levels of measurement involves four types of scale: “Nominal”, “Ordinal”, “Interval” and “Ratio”. This classification has been used widely in medical fields and has accomplished an important role in composition and interpretation of scale. With this classification, levels of measurements appear organized and validated. However, a group theory-like systematization beckons as an alternative because of its logical consistency and unexceptional applicability in the natural sciences but which may offer great advantages in clinical medicine. According to this viewpoint, the Stevens classification is reformulated within an abstract algebra-like scheme; ‘Abelian modulo additive group’ for “Ordinal scale” accompanied with ‘zero’, ‘Abelian additive group’ for “Interval scale”, and ‘field’ for “Ratio scale”. Furthermore, a vector-like display arranges a mixture of schemes describing the assessment of patient states. With this vector-like notation, data-mining and data-set combination is possible on a higher abstract structure level based upon a hierarchical-cluster form. Using simple examples, we show that operations acting on the corresponding mixed schemes of this display allow for a sophisticated means of classifying, updating, monitoring, and prognosis, where better data mining/data usage and efficacy is expected.

## Background

In 1946, S. S. Stevens devised his classification of “levels of measurement” [1], which subsequently has been used widely and has accomplished an important role in composition and interpretation of scales in medical fields. The systematics of levels of measurement seems to have been organized and validated by virtue of this classification. Nevertheless, we believe that an abstract algebra-like interpretation/systematization awaits introduction because of its logical consistency and unexceptional applicability in describing patterns and processes. We conjecture that it offers benefits in clinical medicine, especially, with respect to scales of measurement [2,3].

Thus, in the following, we re-interpret Stevens classification, and endeavour to give it meaning in some abstract algebra-like modelling. There, the most preferred construct is a vector-like structure of various sets of scores based on individual scales and operators that permit changes of score within the set. Additionally, classical datasets that are classified in terms of the Stevens scales of measurement can be mined and combined on a higher abstract structure level based upon a hierarchical-cluster form. To explore this possibility, we provide simple examples to help readers understand this modelling tool.

### §1. Application of group/field of abstract algebra to the various types of scale*s*

Stevens classified the scales of measurement into four scale types [1]; І) “Nominal scale” that uses only labels or numbers (e.g., numbering of football players, blood type, nationality); II) “Ordinal scale” that introduces equality, rank-ordering (e.g., hardness of minerals, grading for efficacy of clinical treatment); III) “Interval scale” that is based on equally quantitative intervals (e.g., temperature as read in centigrade, duration, frequency); and ІV) “Ratio scale” that assumes a ‘zero’ as an origin, equality, rank-order, equality of intervals, and equality of ratios (e.g., absolute temperature, speed of vehicles, and most physical values) that then admit manipulations using the four arithmetic operations.

For І), the “Nominal scale”, there seems to be little room where group theoretical operations apply because within that scale only a labelling scheme is permissible. Although some non-cyclic group might be definable, it seems that little meaning can be attached to operations for this sort of scale.

For II), the “Ordinal scale”, a ranking is realised by introducing a set with an N-graded scoring like ‘1, 2, 3,…, N – 1, N’ (N: positive integer) for a score deficient in (or with no absolute need for) a quantitative character, but not requiring a ‘0’ score according to the Stevens classification. Historically, the “graphic rating scale”, a grading from І to V, was proposed by Hayes and Patterson in 1921 [4], and Freyd in 1923 [5]. However, here, we envisage either operations that decrease the score by ‘1’ in an N-graded graphic scale necessitating a ‘0’, so that {0, 1, 2, 3,…, N – 2, N – 1} establishes the scoring scale, or simply adding the score ‘0’ as in {0, 1, 2, 3,…, N – 2, N – 1, N}. We focus on the former type. Then, for an arbitrary non-negative integer X, the operation giving the remainder of X after division by N, written X (mod N), defines the cyclic group Z_N_ = {0, 1, 2, 3,…, N – 2, N – 1}, where modulo N addition is postulated. With this assumption, given two elements ‘X_j_’ and ‘X_k_’ (X_j_, X_k_ ∈ Z_N_) corresponding for example to the severity of a clinical symptom and/or finding, then composition (denoted by ‘*’) is taken to be modulo N addition; ‘X_j_*X_(j→k)_ = X_k_’ (with X_(j→k)_ ∈ Z_N_). Here ‘X_(j→k)_’ is an operator that produces the change in score, ‘X_j_ → X_k_’ (formally we have ‘X_(j→k)_ = X_j_^-1^*X_k_ = X_k_ – X_j_’). Then, all scores ‘X_j_’s and operators ‘X_(j→k)_’ are composable within a single Abelian modulo additive group ‘Z_N_’, where ‘X_j_*X_k_ = X_k_*X_j_’ holds, at least, in terms of operation ‘*’. Thus a patient’s state corresponding to a certain illness or disease can be changed through the application of a single operation determined by the two elements belonging to ‘Z_N_’ [6,7] representing the previous and current state of the patient. A simple example is presented in Appendix A.

If a state of maximum severity is present, then the antithesis for any given disease Y is the ideal healthy state E_Y_ = [0|0|0|0|0|…], the combination of all scores being ‘0’ and represented by the identity element for group Y = {Z_N_^×n^, *}. Here, Y is the n-fold Cartesian product of ‘Z_N_’ (n: the number of components) that comprises all possible assessments related to each state of a given disease, for instance, ‘hypertension’, ‘hyperglycaemia’, ‘diabetes mellitus’, ‘acute pancreatitis’, ‘systemic lupus erythematosus’, and ‘cerebral artery stroke’. If in addition composition is given by modulo ‘N’ arithmetic, prime numbers (e.g., N = 7) are preferable [8,9] and considerable parts of components could be overlapping among individual diseases as was mentioned in our previous reports [6,7]. Note that, in practice, equal increments within a grading scheme are not always postulated. Nevertheless, the scale represented by this Abelian modulo additive group ‘Z_N_^×n^’ will be called a “modular scale”. However, it may be an atypical case (partially weakened example) of a “Ratio scale” (type ІV) without the strict requirement for equal calibration. Indeed, there are such scales because, like the ‘TNM classification (with a ‘T0’ entry) for malignant tumours’ [7,10], grades for scoring are determined for example according to histological characteristics, selection of treatment, and prognosis, having no strict linearity in scale, but which might be regarded as an “modular scale”. Based upon these results, for instance, the following are considered composable; Abelian modulo additive group Y_1_ = {Z_7_, *} for ‘hypertension’, Y_2_ = {Z_7_, *} for ‘hyperglycaemia’, Y_3_ = {Z_7_, *} for ‘diabetes mellitus’, Y_4_ for ‘acute pancreatitis’, Y_5_ for ‘systemic lupus erythematosus’, Y_6_ for ‘cerebral artery stroke’, Y_all_ = {Z_7_ × Z_7_ × Z_7_ × …, *} = {Z_7_^×n^, *} (n: the number of components) for an entire body, and Y_7_ = {Z_8_ × Z_4_ × Z_2_ × Z_2_, *} for the ‘TNM classification (with a ‘T0’ entry) for malignant tumours’ [7,10]. Additionally, these are treatable without exception within the abstract algebraic theory. For this case, an equal calibration for severity may have unbeneficial outcomes if used in clinical treatments. However, for ‘delirium’, ‘chronic liver dysfunction’, ‘acute pancreatitis’, and ‘diabetes mellitus’, for example, total scores based on equal calibration are desirable to assess disease severity.

For III), the “Interval scale”, differences in quantities are allowed. An example is ‘periods of time’ or ‘duration’, which, although can be measured with ratio scales, enables one period to be double another when compared. The same is true of ‘temperature’. If parameters ‘X_j_’ and ‘X_l_’ ∈ R (the continuous real number line) have ranges

(i) -∞<X<+∞

we can consider an operator ‘X_k_’ that causes changes from ‘X_j_’ to ‘X_l_’, and introduce a binary operation, denoted ‘◦’, where ordinal addition and its inverse, subtraction, are assumed;

(ii) $$X_{j}\circ X_{k}=X_{j}+X_{k}=X_{l}\;\left(j,k,l;\mathrm{session}\;\mathrm{numbers}\right)$$

In this regard, as for ‘X_j_’, it can also be expressed as a sum of an integer part and a decimal part,

(iii) $$X_{j}=1m_{j}+c_{j}$$

(m_j_ = [X_j_], c_j_ = X_j_ - [X_j_], ‘0 ≤ c_j_ < 1’; ‘[X]’ is the floor function meaning the highest integer below ‘X’). Similarly,

(iv) $$X_{k}=1m_{k}+c_{k}\left(m_{k}=\left[X_{k}\right],c_{k}=X_{k}-\left[X_{k}\right],‘0\leq c_{k}<1’\right)$$

(v) $$X_{l}=1m_{l}+c_{l}\;\left(m_{l}=\left[X_{l}\right],c_{l}=X_{l}-\left[X_{l}\right],‘0\leq c_{l}<1’\right)$$

‘1’ is a ‘unit length’ of the respective values. Thus, (iii) - (v) can be redefined using the unit length ‘1’ as an interval scale,

(vi) $$\begin{array}{l} X_{j}\circ X_{k}=\left(1m_{j}+c_{j}\right)+\left(1m_{k}+c_{k}\right)\\ \;=1\left(m_{j}+m_{k}\right)+\left(c_{j}+c_{k}\right)=1m_{l}+c_{l}\\ \;=X_{l} \end{array}$$

There exists an identity element ‘X_0_’ (=0) that satisfies ‘X_j_ ◦X_0_ = X_0_ ◦X_j_ (=X_j_ + X_0_ = X_j_ + 0) = X_j_’. Additionally, the inverse element is ‘X_j_^-1^ = -X_j_’ satisfying ‘X_j_^-1^◦X_j_ = X_j_ ◦X_j_^-1^ = X_j_ + X_j_^-1^ = X_j_ - X_j_ = X_0_ (=0)’.

Naturally, commutativity and associativity are satisfied. Let U be the set that comprises all ‘X_j_’s, i.e., U ≡ {X_j_ | X_j_ ∈ R}. Because ‘X_j_ , X_k_, X_j_ ∈ set U, the closure law holds. Therefore, this operation defines a group U = {X_j_, ◦} [2,3]. “Body temperature readings”, “clock time for the onset of sleep within a day” and “clock time for the onset of drip infusion within a day” are definable in this scale. Examples of the first two are provided in Appendix B. By making use of this procedure, the differences between quantitative values and operators are eliminated, and both can be regarded as elements belonging to a single group U. Moreover, a collection of additive Abelian groups U_1_ ≡ {X_1j_ | X_1j_ ∈ R (deg C)} based on an individual’s clinical values can be described as, as for example U_1_ = {X_1j_, ◦} for “body temperature readings”, and U_2_ ≡ {X_2j_ | X_2j_ ∈ R (/24 hrs)} and U_2j_ = {X_2j_, ◦} for “clock time for the onset of sleep within a day”, U_3_ ≡ {X_3j_ | X_3j_ ∈ R (/24 hrs)} and U_3j_ = {X_3j_, ◦} for “clock time for the onset of drip infusion within a day”,…, U_N_ = {X_Nj_, ◦},…, (N: natural number). Those are considered readily treatable and recordable within an abstract algebraic context.

For IV), the “Ratio scale”, the ‘administration of medicine (with strict dosage regimes)’ and ‘International Statistical Classification and Health Related Problems’ [11] were given as examples in our previous report [6,7]. Essentially, for this scale, because the four arithmetic operations are possible, ‘rings’ and ‘fields’ in abstract algebra are applicable so long as composition is given by modulo ‘N’ arithmetic with ‘N’ a prime. Although there could be scope where the four modulo arithmetic operations (denoted by ‘†’ in ‘X_j_†X_k_ = X_l_’) are applicable in assessment scoring in clinical medicine, it might be preferable at this stage to confine the application of ratio scales to just modulo N addition ‘*’ collectively for ‘†’, similar in manner as established in Appendix A. For the example given in Appendix A, the difference in interpretation is the presence/absence of an equal calibration.

Whereas the scale of ‘TNM classification for malignant tumours’ [10] was regarded as an example of an “Ordinal scale”, some of the scales defined as “Ratio scales” at initial glance should be regarded as “Ordinal scales” accompanied with ‘0’. It might be contentious whether clinical assessments performed using superficial scales based on the four arithmetic operations could have sufficient validity in clinical treatments or clinical research.

Nevertheless, other clinical scales range over a semi-open continuous interval like ‘0 ≤ X < +∞’ (X: real number), such as ‘blood concentration of white blood cells: [WBC] (/mm^3^)’, and ‘administration of a certain drug like lithium carbonate: [Li^+^] (mEq/l), sodium: [Na^+^] (mEq/l), calcium: [Ca^++^] (mg/dl), chloride: [Cl^-^] (mEq/l) and bicarbonate: [HCO_3_^-^] (mEq/l)’. Also, there are clinical scales whose ranges are the open interval like ‘-∞ < X < +∞’ (X: real number); ‘Anion gap [AG] = [Na^+^] - ([Cl^-^] + [HCO_3_^-^]) (reference range for blood tests: 12 ± 2 mEq/l)’ and ‘Base excess [BE] (reference range for blood tests: 0 ± 2 mmol/l)’. However, both can be treated using the notion of ‘field’ because those values are real numbers where all four arithmetic operations are included, with the exception of division by zero. Thus, the above clinical values could be definable over a ‘field’. In this regard, we assume a rule that each unit like ‘mEq/l’ accompanies the value automatically with the results of operations regardless of types of operation among the four arithmetic operations (Note that there are cases when units vanish as when ratios are taken ‘mEq/mEq (unitless)’ or displayed in reciprocal form like ‘l/mEq’). Examples for ‘[WBC] (/mm^3^)’, ‘[Na^+^] (mEq/l)’ are presented in Appendix C.

In this case, we consider a set V and assume that ‘#’ means one of ‘addition, subtraction, multiplication, and division’ collectively; thus, ‘X_j_ # X_k_ = X_l_ (∈V), where ordinal arithmetic calculations are performed excluding of course division by zero.

For set V, addition is commutative: X_j_ + X_k_ = X_k_ + X_j_, and associative: (X_j_ + X_k_) + X_l_ = X_j_ + (X_k_ + X_l_). As for multiplication, set V meets the conditions of a ‘monoid’ [2,3]. Associativity: (X_j_ × X_k_) × X_l_ = X_j_ × (X_k_ × X_l_), with Left and Right Distributivity: X_j_ × (X_k_ + X_l_) = X_j_ × X_k_ + X_j_ × X_l_, (X_j_ + X_k_) × X_l_ = X_j_ × X_l_ + X_k_ × X_l_. A nonzero Identity X_0_ (=1) for multiplication exists. The Inverse ‘X_j_^-1^ = 1/X_j_’ satisfies ‘X_j_ × X_j_^-1^ = X_j_^-1^ × X_j_ = X_0_ (=1)’. For division, ‘X_j_/X_k_ = X_j_ × X_k_^-1^ = 1’ is definable except for division by zero. Therefore, we can confirm that set V is a ‘field’. It can be expressed as V = {X_j_, #} or V = {X_j_ | X_j_ ∈ R}.

Furthermore, different fields based on different sets of clinical values can be described as follows: field V_1_ ≡ {X_1j_ | X_1j_ ∈ R (/mm^3^)} and V_1_ = {X_1j_, #} for “blood concentration of white blood cells: [WBC] (/mm^3^)”, field V_2_ ≡ {X_2j_ | X_2j_ ∈ R (mEq/l)} and V_2_ = {X_2j_, #} for “administration of a certain drug like lithium carbonate: [Li^+^] (mEq/l)”, field V_3_ ≡ {X_3j_ | X_3j_ ∈ R (mEq/l)} and V_3_ = {X_3j_, #} for “sodium: [Na^+^] (mEq/l)”, field V_4_ ≡ {X_4j_, #} for calcium: [Ca^++^] (mg/dl), field V_5_ for chloride: [Cl^-^] (mEq/l), field V_6_ for ‘Anion gap [AG] (mEq/l)’, field V_7_ for ‘Base excess [BE] (mmol/l)’,…, V_N_,…, (N: natural number). For each, an independent abstract algebraic treatment is possible as for ordinal abstract algebra.

### §2. A vector-like notation using group/field operations belonging to a single set

By making use of all types of scales of measurement, we propose a vector-like expression of a patient’s state (denoted ‘R_j_’, j = 1, 2, 3,…: number of sessions), where the mixed expression and its totality of operations that could be performed belong to a single set R. Because of the possible variety of operation rules, the genuine use of this set may be unwieldy at this stage.

Partially based upon our previous description [6,7], let us define ‘R_j_’ to be a vector of five clinical values,

R_j_ = [severity for depression (within modulo 7 arithmetic) | clock time for the onset of sleep (/24 hrs) | blood concentration of white blood cell [WBC] (/mm^3^) | blood concentration of [Na^+^] (mEq/l)| a certain value (a certain operational unit)],

(vii) $$\begin{array}{l} =\left[X_{\left(j\right)1}\right)\left(\mathrm{mod}7\right)\;|\;X_{\left(j\right)2}\left(/24\;\mathrm{hrs}\right)|\;X_{\left(j\right)3}\left(/{\mathrm{mm}}^{3}\right)\\ \;|\;X_{\left(j\right)4}\left(\left(\mathrm{mEq}/l\right),|,X_{\left(j\right)5},\left(\ldots \right)\right] \end{array}$$

Next, suppose the patient’s state ‘R_j_’ changes to ‘R_j+1_’ effected by operator ‘R_(j→j+1)_’; we denote by ‘◊’ the binary composition composed of the product of compositions for each component. Three possible states are:

$$\begin{array}{l} R_{1}=\left[\left(X_{\left(1\right)1}=\right)\;2\;\left(\mathrm{mod}7\right)\;|\;\left(X_{\left(1\right)2}=\right)\;21\;\left(/24\;\mathrm{hrs}\right)\;|\;\left(X_{\left(1\right)3}=\right)5000\right)\\ \;\left(/{\mathrm{mm}}^{3}\right)|\;\left(X_{\left(1\right)4}=\right)\;145\left(\left(\mathrm{mEq}/l\right)|X_{\left(1\right)5}\left(\ldots \right)\right] \end{array}$$

$$\begin{array}{l} R_{2}=\left[\left(X_{\left(2\right)1}=\right)\;5\;\left(\mathrm{mod}7\right)\;|\;\left(X_{\left(2\right)2}=\right)\;19.5\;\left(/24\;\mathrm{hrs}\right)|\;\left(X_{\left(2\right)3}=\right)18000\right)\\ \;\left(/{\mathrm{mm}}^{3}\right)|\;\left(X_{\left(2\right)4}=\right)\;128\left(\left(\mathrm{mEq}/l\right)|X_{\left(2\right)5}\left(\ldots \right)\right]\;, \end{array}$$

$$\begin{array}{l} R_{3}=\left[\left(X_{\left(3\right)1}=\right)\;3\;\left(\mathrm{mod}7\right)\;|\;\left(X_{\left(3\right)2}=\right)\;22\;\left(/24\;\mathrm{hrs}\right)|\;\left(X_{\left(3\right)3}=\right)\;7000\right)\\ \;\left(/{\mathrm{mm}}^{3}\right)|\;\left(X_{\left(3\right)4}=\right)\;158\;\left(\left(\mathrm{mEq}/l\right)|X_{\left(3\right)5}\left(\ldots \right)\right]. \end{array}$$

For the 1st component, ‘X_(1)1_’,‘X_(2)1_’, and ‘X_(3)1_’, modulo 7 arithmetic (addition) is used. For the 2nd components, ‘X_(1)2_, X_(2)2_, X_(3)2_’, operations of Abelian addition are used. For the 3rd component, ‘X_(1)3_, X_(2)3_, X_(3)3_’, 4th ‘X_(1)4_, X_(2)4_, X_(3)4_’, the four arithmetic operators (those operations denoted by ‘#’) are required, and for the 5^th^, ‘X_(1)5_, X_(2)5_, X_(3)5_’, a certain operational unit is postulated. In the following examples, only addition/subtraction is presented; naturally, multiplication/division is also considered permissible.

With related operators R_(1→ 2)_ = [X_(1→ 2)1_(mod 7) | X_(1→ 2)2_(/24 hrs)| X_(1→ 2)3_(/mm^3^)| X_(1→ 2)4_(mEq/l)|X_(1→ 2)5_(…)], and R_(2→ 3)_ = [X_(2→ 3)1_(mod 7) | X_(2→ 3)2_(/24 hrs)| X_(2→ 3)3_(/mm^3^)| X_(2→ 3)4_(mEq/l)|X_(2→ 3)5_(…)]

Then, using results in Appendix D, ‘R_(1→2)_’ and ‘R_(2→3)_’ from the three states given above are as follows:

$$\begin{array}{c} R_{\left(1\to 2\right)}=\left[3\;\left(\mathrm{mod}7\right)\;|-1.5\left(/24\;\mathrm{hrs}\right)|13000\left(/{\mathrm{mm}}^{3}\right)|\right)\\ \;-17\left(\mathrm{mEq}/l\right)\left[|X_{(1\to 2)5}\left(\ldots \right)\right] \end{array}$$

$$\begin{array}{c} R_{\left(2\to 3\right)}=\left[5\left(\mathrm{mod}7\right)\;|2.5\left(/24\;\mathrm{hrs}\right)|-11000\left(/{\mathrm{mm}}^{3}\right)|\right)\\ \;30\left(\mathrm{mEq}/l\right)|\left(X_{(2\to 3)5}\left(\ldots \right)\right] \end{array}$$

Thus, we confirm the relation

(viii) $$R_{1}◊R_{\left(1\to 2\right)}◊R_{\left(2\to 3\right)}=R_{3}$$

Details are illustrated in Appendix E.

Note that, in general, there exists an identity ‘E (=R_0_) = [0 (mod 7)| 0 (/24 hrs)| 0 (/mm^3^)| 0 (mEq/l) | X_0_ (…)]’ such that ‘R_j_◊E = E◊R_j_ = R_j_’. Additionally, there exists an inverse for any ‘R_j_’, ‘R_j_^- 1^ = [X_(j)1_^- 1^(mod 7) | X_(j)2_^- 1^(/24 hrs)| X_(j)3_^- 1^(/mm^3^)| X_(j)4_^- 1^(mEq/l)|X_(j)5_^- 1^(…)] = [7–X_(j)1_(mod 7) | 24 - X_(j)2_(/24 hrs)| - X_(j)3_(/mm^3^)| - X_(j)4_(mEq/l)|X_(j)5_^- 1^(…)]’ that satisfies ‘R_j_^-1^◊R_j_ = R_j_◊R_j_^-1^ = E’. However, commutativity, ‘R_j_◊R_k_ = R_k_◊R_j_’ and associativity, ‘(R_j_◊R_k_)◊R_l_ = R_j_◊(R_k_◊R_l_)’ are not satisfied. Here, we assume that operators acting on ‘R_j_’s should be performed from left to right, that is, from R_1_ to R_m_ (m; number of session for assessment). They should not be applied between ‘R_j_’s. For any assortment of ‘R_j_’s with scales of measurement among types I)–IV), a single set R = {R_j_| X_(j)1_ × X_(j)2_ × X_(j)3_ × X_(j)4_ × X_(j)5_} (‘×’ means products among groups and fields) using a vector-like notation for the scoring of patient states can be structured where all possible assessments and/or clinical findings of the patient and treatment are included. The general form is the n-fold product; set R = {R_j_| X_(j)1_ × X_(j)2_ × X_(j)3_ × X_(j)4_ × …×X_(j)(n-2)_ × X_(j)(n-1)_ × X_(j)n_} (n; the number of components).

**Figure 1:**
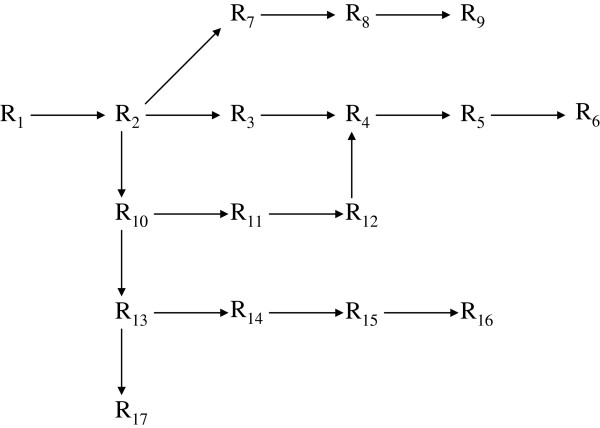
**Example of a tree composed of a data set and ordinal arrows.** The tree represents changes of states between 17 ‘R_j_’s data elements. In ordinal existing methods, data are only a collection of results that are not directly combined; manipulations of parts of the data are defined separately. The arrows merely indicate a change from one state ‘R_j_’ to another ‘R_k_’ and have no specific operational sense.

**Figure 2:**
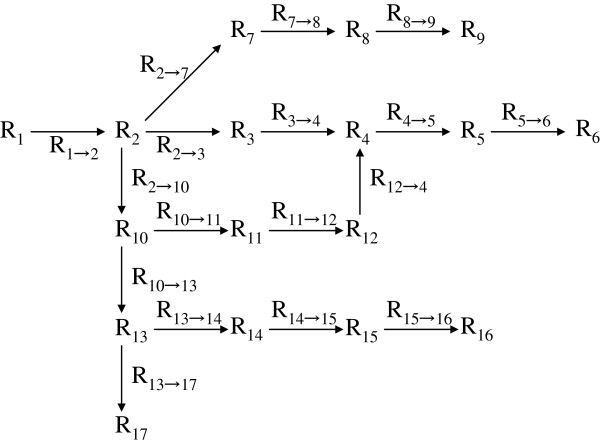
**Scheme of the tree with arrows labelled by operators.** Arrows are interpreted as operators ‘R_(j→k)_’s that could be regarded as ordinal elements ‘R_j_’s belonging to a single set R. Each operator that changes ‘R_j_ to R_k_’ can be traced and its degree for each session identified from initial and final states. The final states (R_6_, R_9_, R_16_ and R_17_) can be traced back to any initiating state ‘R_1_’ by performing an appropriate sequence of ‘R_(j→k)_’s.

**Figure 3:**
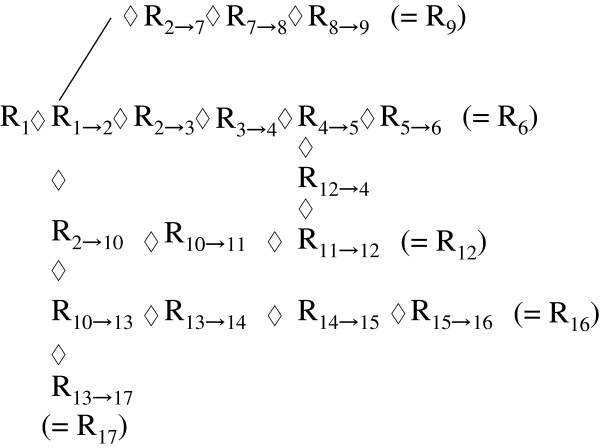
**“Operational tree”—the compositional scheme using symbol ‘◊’.** The tree of Figure 2 is re-illustrated using composition symbol ‘◊’, where the operators are assumed to belong to the single set R. By admitting algebraic correspondences, this compositional scheme could potentially provide better data mining/data usage.

As for the possible application to better data mining or data usage from the viewpoint of our reinterpretation, we provide a simple example that may help readers to follow an outline of the argument. Consider an example of 17 states “R_1_, R_2_, …, R_17_” (∈set R) each with four component (‘n = 5’) and arrows (only symbols) that indicate the possible changes among the ‘R_j_’s, as displayed in Figure 1. The scheme covers the notation of our model, and also that of existing methods where (possible) results of data, ‘R_j_’s, are not combined directly with each other in the sense of operations. Then, the arrows could be re-displayed according to our concepts as operators ‘R_(j→k)_ that can be regarded as elements ‘R_j_’ belonging to a set R as in Figure 2. In ordinal data sets, the ‘R_j_’s are merely a collection of values and the arrows in Figure 1 are only marks. However, in our interpretation, all ‘R_j_’s and ‘R_(j→k)_’s are elements of a single set R subject to axioms of an abstract algebra as indicated using composition symbol ‘◊’ in Figure 3. There, the changes ‘from R_j_ to R_k_’ can be traced at each session. Displayed in this way, Figure 3 represents an “operational tree” that could offer potential for better data mining/data usage through a more generalized/concise treatment (e.g., withdrawing/recording correspond to schemes in Figure 3) that might be permissible. Practical improvements for efficacy, however, will need future investigations.

Here, consider the scenario of Figure 1 where from an initial value ‘R_1_’ there are four outcomes ‘R_6_’, ‘R_9_’, ‘R_16_’, and ‘R_17_’ containing nodes at ‘R_2_’ ‘R_4_’ ‘R_10_’ ‘R_12_’ and ‘R_13_’. By making use of our previous examples ‘R_1_ - R_3_’, the next simplest examples with ‘n (component number) = 5’ can be confirmed easily:

$$\begin{array}{c} R_{10}=\left[0\;\left(\mathrm{mod}7\right)\;|17\;\left(/24\;\mathrm{hrs}\right)|9000\;\left(/{\mathrm{mm}}^{3}\right)\right)\\ \;|130\;\left(\mathrm{mEq}/l\right)\left[|X_{\left(10\right)5}\left(\ldots \right)\right) \end{array}$$

$$\begin{array}{c} R_{11}=\left[6\;\left(\mathrm{mod}7\right)\;|20\;\left(/24\;\mathrm{hrs}\right)|20000\;\left(/{\mathrm{mm}}^{3}\right)\right)\\ \;|149\;\left(\mathrm{mEq}/l\right)\left[|X_{\left(11\right)5}\left(\ldots \right)\right) \end{array}$$

$$\begin{array}{c} R_{12}=\left[4\;\left(\mathrm{mod}7\right)\;|23\;\left(/24\;\mathrm{hrs}\right)|6000\;\left(/{\mathrm{mm}}^{3}\right)\right)\\ \;|140\;\left(\mathrm{mEq}/l\right)\left(|X_{\left(12\right)5}\left(\ldots \right)\right] \end{array}$$

$$\begin{array}{c} R_{13}=\left[1\;\left(\mathrm{mod}7\right)\;|18\;\left(/24\;\mathrm{hrs}\right)|5000\;\left(/{\mathrm{mm}}^{3}\right)\right)\\ \;|135\;\left(\mathrm{mEq}/l\right)\left(|X_{\left(13\right)5}\left(\ldots \right)\right] \end{array}$$

$$\begin{array}{c} R_{17}=\left[2\;\left(\mathrm{mod}7\right)\;|23.5\;\left(/24\;\mathrm{hrs}\right)|3000\;\left(/{\mathrm{mm}}^{3}\right)\right)\\ \;|150\;\left(\mathrm{mEq}/l\right)\left(|X_{\left(17\right)5}\left(\ldots \right)\right] \end{array}$$

Following these results, the next relations, according to the tree in Figure 3, can be obtained for instance:

$$R_{1}◊R_{\left(1\to 2\right)}◊R_{\left(2\to 10\right)}◊R_{\left(10\to 11\right)}◊R_{\left(11\to 12\right)}=R_{12}$$

$$R_{1}◊R_{\left(1\to 2\right)}◊R_{\left(2\to 10\right)}◊R_{\left(10\to 13\right)}◊R_{\left(13\to 17\right)}=R_{17}$$

The operator expressions are evaluated in Appendix F.

Similarly, the next sequences are definable in principle,

$$R_{1}◊R_{\left(1\to 2\right)}◊R_{\left(2\to 3\right)}◊R_{\left(3\to 4\right)}◊R_{\left(4\to 5\right)}◊R_{\left(5\to 6\right)}=R_{6}$$

$$R_{1}◊R_{\left(1\to 7\right)}◊R_{\left(7\to 8\right)}◊R_{\left(8\to 9\right)}=R_{9}$$

$$R_{12}◊R_{\left(12\to 4\right)}=R_{4}$$

$$R_{1}◊R_{\left(1\to 2\right)}◊R_{\left(2\to 10\right)}◊R_{\left(10\to 13\right)}◊R_{\left(13\to 14\right)}◊R_{\left(14\to 15\right)}◊R_{\left(15\to 16\right)}=R_{16}$$

In general, we denote a node divergence ‘R_a_ to R_b_ (=R_a_◊R_(a→b)_ = R_b_)’ and ‘R_a_ to R_c_ (=R_a_◊R_(a→c)_ = R_c_)’ as ‘R_a_[(◊R_(a→b)_)(◊R_(a→c)_)]’ (a,b,c: non-negative integers); here ‘( )( )( )…’ meaning simple juxtaposition. All paths belonging to the operational tree of Figure 3 can then be described/recorded, for instance, as the sequence

(ix) $$\begin{array}{l} R_{1}◊R_{\left(1\to 2\right)}\left[\left(◊R_{\left(2\to 3\right)}◊R_{\left(3\to 4\right)}◊R_{\left(4\to 5\right)}◊R_{\left(5\to 6\right)}\right)\left(◊R_{\left(2\to 7\right)}◊R_{\left(7\to 8\right)}\right)\right)\\ \;(\left(◊R_{\left(8\to 9\right)}\right)(◊R_{\left(2\to 10\right)}[\left(◊R_{\left(10\to 11\right)}◊R_{\left(11\to 12\right)}◊R_{\left(12\to 4\right)}\right)\\ \;\left(◊R_{\left(10\to 13\right)}\left[\left(◊R_{\left(13\to 14\right)}◊R_{\left(14\to 15\right)}◊R_{\left(15\to 16\right)}\right)\left(◊R_{\left(13\to 17\right)}\right)])]\right)\right] \end{array}$$

To display for easy recognition, for example, end states like ‘R_6_, R_9_, R_16_, and R_17_’ and divergence point ‘R_4_’ a notation ‘(=R_6_), (=R_9_), (=R_16_) and (=R_17_)’ might be considered. Hence,

(x) $$\begin{array}{l} R_{1}◊R_{\left(1\to 2\right)}\left[\left(◊R_{\left(2\to 3\right)}◊R_{\left(3\to 4\right)}◊R_{\left(4\to 5\right)}◊R_{\left(5\to 6\right)}\left(=R_{6}\right)\right)\left(◊R_{\left(2\to 7\right)}\right)\right)\\ \;(\left(◊R_{\left(7\to 8\right)}◊R_{\left(8\to 9\right)}\left(=R_{9}\right)\right)(◊R_{\left(2\to 10\right)}[\left(◊R_{\left(10\to 11\right)}◊R_{\left(11\to 12\right)}\right)\\ \;◊\left(R_{\left(12\to 4\right)}\left(=R_{4}\right)\right)\left(◊R_{\left(10\to 13\right)}\left[\left(◊R_{\left(13\to 14\right)}◊R_{\left(14\to 15\right)}\right)\right)\right)\\ \;◊\left(\left(\left(R_{\left(15\to 16\right)}\left(=R_{16}\right)\right)\left(◊R_{\left(13\to 17\right)}\left(=R_{17}\right)\right)])]\right)\right] \end{array}$$

Moreover, composition with an operator as in operating on ‘R_(3 → 4)_[(◊R_(4 → 5)_ ◊ R_(5 → 6)_)(◊R_(4 → 8)_ ◊ R_(8 → 9)_ ◊ R_(9 → 10)_)(◊R_(4 → 15)_ ◊ R_(15 → 16)_) …] from the left-hand side by ‘R_3_’. The subsequent result can be expressed in accordance with the single scheme presented in Figure 3,

(xi) $$\begin{array}{l} R_{3}◊R_{\left(3\to 4\right)}\left[\left(◊R_{\left(4\to 5\right)}◊R_{\left(5\to 6\right)}\right)\left(◊R_{\left(4\to 8\right)}◊R_{\left(8\to 9\right)}◊R_{\left(9\to 10\right)}\right)\right)\\ \;\left(\left(◊R_{\left(4\to 15\right)}◊R_{\left(15\to 16\right)}\right)\ldots \right]=R_{6},R_{10},R_{16},\ldots \end{array}$$

Note that the above descriptions (ix)–(xi) express one-to-many functionality. However, we think that these formulae are the algebra equivalent to the single operational tree as exemplified by Figure 3. These play the algebraic role in composite record-keeping in applied fields such as medicine. In this formalism, any possible result ‘R_j_’ (∈set R) is obtained and traceable from any state ‘R_k_’ under operations involving a plurality of elements belonging to a single set R.

**Figure 4:**
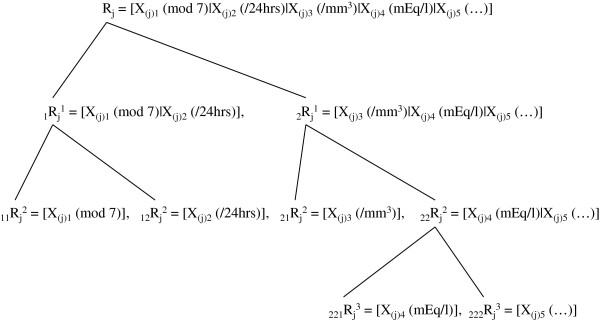
**Systematization of “hierarchical clusters”.** A hierarchical cluster is defined as the necessary class of subsets needed to decompose the set to single units. The top level is the entire dataset and that is always decomposable into base units.

**Figure 5:**
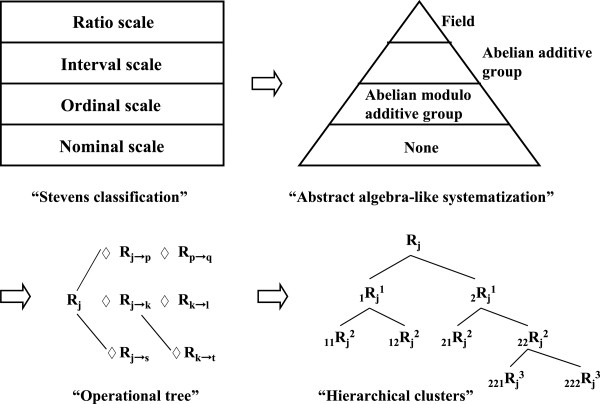
**Schemes for data mining and combination in some higher abstract structure level.** First, the classical dataset, classified by the four types of scales of measurement (Stevens classification), is re-interpreted as a group/field-like operational structure. Second, a vector ‘R_j_’ is defined that is composed as the product of each type of operation for all datasets other than for those classified as “Nominal scale”. Third, the ‘R_j_’s are constituted as an “operational tree”. Fourth, ‘R_j_’ with any arbitrary j (j = 1, 2,…, m) could be mined and combined as “hierarchical clusters”.

Additionally, we can include data mining in a more symbolic/abstract way as follows. For an arbitrary j (j = 1, 2, 3,…, m), a hierarchical-cluster-like expression can be defined [12]. For instance, if a partition of R_j_ is a set of subsets H = {_1_R_j_, _2_R_j_, _3_R_j_,…, _r_R_j_} such that (1) R_j_ ∈ H; (2) for all single sets _s_R_j_ in R_j_, _s_R_j_ ∈ H; and (3) ‘_s_R_j_ ∩ _t_R_j_ ∈ {ϕ, _s_R_j_, _t_R_j_}’ for all s ≠ t = 1, 2,…, r. That is, condition (3) means that either any two clusters ‘_s_R_j_ and _t_R_j_’ are disjoint, or one cluster is contained entirely inside the other, and every individual R_j_ is contained in at least one cluster larger than itself. Note that if ‘_s_R_j_ ∩ _t_R_j_ = ϕ’ for all s ≠ t, then the hierarchy becomes a partitioning. Henceforth, reference to a hierarchy implies that ‘_s_R_j_ ∩ _t_R_j_ = ϕ’ for at least one set of (s, t) values. In the previous example (vii), ‘R_j_’ could be expressed in hierarchical-cluster notation where there are eight clusters (and relabeling within clusters) as shown in Figure 4. If R_j_ comprises ‘_1_R_j_^1^ and _2_R_j_^1^’, the first level of hierarchy, ‘R_j_ = _1_R_j_^1^ ∪ _2_R_j_^1^’ holds. At the second level, ‘_1_R_j_^1^ = _11_R_j_^2^ ∪ _12_R_j_^2^’ = [X_(j)1_ (mod 7) | X_(j)2_ (/24 hrs)], ‘_2_R_j_^1^ = _21_R_j_^2^ ∪ _22_R_j_^2^’ = [X_(j)3_ (/mm^3^) | X_(j)4_ (mEq/l)|X_(j)__5_ (…)], whereas at the third level, ‘_22_R_j_^2^ = _221_R_j_^3^ ∪ _222_R_j_^3^’ = [X_(j)4_ (mEq/l)|X_(j)__5_ (…)], _221_R_j_^3^ = [X_(j)4_ (mEq/l)], _222_R_j_^3^ = [X_(j)5_ (…)] (Figure 4). Hence we obtain the complete set R_j_ = {X_(j)1_, X_(j)2_, X_(j)3_, X_(j)4_, X_(j)5_} = [X_(j)1_ (mod 7) | X_(j)2_ (/24 hrs) | X_(j)3_ (/mm^3^) | X_(j)4_ (mEq/l) | X_(j)5_ (…)]. A hierarchy has additional levels as necessary to reach single units at its base [12]. The top level is the entire dataset ‘R_j_’ and that is always composable using base units. That is, arbitrary ‘R_k_’ and ‘R_1_’ can be combined into a single dataset as with ‘R_k_ = [X_(k)1_| X_(k)2_ |…| X_(k)a_]’ and ‘R_1_ = [X_(1)1_| X_(1)2_ |…| X_(1)b_]’, ‘{R_k_, R_1_} =R_j_ [X_(j)1_| X_(j)2_ |…| X_(j)a_ | X_(j)a+1_| X_(j)a+2_ |…| X_(j)a+b_] ’ (a,b; positive integers). In this way, classical datasets that are classified in the Stevens scales of measurement could be mined and combined on a higher abstract structure level. To help better understand the concept, a sequence of schemes illustrating the principles of our model is presented in Figure 5.

Subject to future improvements, we envisage that this compact description is versatile to provide better data mining/data usage than from existing methods, although a final version is far from complete at this early stage.

### §3. Supplementary suggestions and limitations

If the four arithmetic operations are appropriate in handling the values from clinical assessments, representation by “Ratio scales” (in some cases, the “modular scale” with suitable modulo number previously mentioned) might be effective in describing the clinical treatments or studies. The “Numerical rating scale (NRS)” with range ‘0–10’ [13,14] illustrates the point where the modulo 11 additive group ‘Z_11_’ arises as a natural modular scale. In contrast, similar approaches might be difficult for a “visual analogue scale” [15,16] where values could take any real number.

Whereas rating scales systemized as abstract algebra-like form may enable a more generalized/sophisticated understanding, establishing a link between fields of clinical medicine and abstract algebra, and mixed states and operators in vector-like notation as in (vii)–(xi), does not always assure more concise manipulations. A mixed treatment as exemplified in (vii)–(xi) might not always yield optimal results at present. In general, combining group and field-like structures within ‘R_j_’ may cause some confusion in handling the ‘R_j_’s although benefits accrue through operational compliance and convenience in dealing with the abstract algebra. For description and records, a vector-like definition ‘R_j_’ may not always be advantageous in which only the four types I)–IV) are used (particularly for ‘I)’, the “nominal scale”, where systematization of operation seems to be impossible). Nevertheless, we infer that in the handling of operations in mixed-notation like ‘R_j_’, the classification and synthesis of scales of measurement in some group/field-like form may be devised in a more rigorous methodology in future improvements.

That apart, similar, redundant, and obscure components may have been incorporated into the ‘R_j_’s description without discretion. The ‘R_j_’ in such instances loses validity and versatility in terms of a concise composition of scales. This is considered to result from the fact that a total state of a certain disease or a condition of a patient is not always composable or describable via the combination of partial components. This implies that a larger number of components is not always desirable for assessment or rating scales.

Unfortunately, almost all current assessment scales in medicine are handled as if they were ratio scales although almost all are just ordinal scales. That might introduce considerable futility and/or waste of scientific resources. As previously indicated, some clinical scales (e.g., TNM classification) should be represented as an ordinal scale accompanied by ‘0’ with no absolute need for a quantitative calibration (modular scale). Although a combination composed of entirely ratio scales seems to be difficult or impossible, we believe at least that appropriate operational structures (e.g., group, field) should always be selected that satisfied the conditions in instances like composition of scale, analysis, and interpretation of the results. These structures must be recognized clearly by users per each assessment to avoid misestimation, overconfidence, and complacency in scales.

## Conclusions

The Stevens classification of scales of measurement can be re-interpreted and modelled as some abstract algebra-like systematization. Moreover, a vector-like notation using mixed types of operations and a hierarchical structure-like systematization are possible enabling a sophisticated means to classify, update, monitor, and forecast patient treatments. Better data mining/data usage and efficacy is expected and will be considered in future studies.

## Appendix

### Appendix A

Using ‘N = 5’ for the scale of a certain symptom or clinical finding with set Z_5_ ≡{0, 1, 2, 3, 4}, we suppose ‘X_1_ = 1’ (∈ set Z_5_) for the initial state and ‘X_2_ = 4’ (∈ set Z_5_) for the final state. Expressed as ‘X_1_*X_(1→2)_ = X_2_’, the change can be determined as ‘X_(1→2)_ = X_2_ – X_1_ (mod 5) = 4 – 1 (mod 5) = 3 (mod 5) (∈ Z_5_)’.

### Appendix B

Suppose ‘the body-temperature thermometer’ (deg C; degree Celsius) changes from ‘T_1_ = 36.7 (deg C)’ to ‘T_2_ = 35.1 (deg C)’. Because ‘T_1_ ◦T_(1→2)_ = T_2_’, an operator part is calculated as ‘T_(1→2)_ = T_2_ - T_1_ = 35.1 - 36.7 (deg C) = - 1.6 (deg C)’. For an another example, when there are two clock times for the onset of sleep ‘t_1_ = 21 (/24 hrs)’ and ‘t_2_ = 19.5 (/24 hrs)’, the operator part is determined as ‘t_(1→2)_ (/24 hrs) = t_2_ - t_1_ (/24 hrs) = 19.5 - 21 (/24 hrs) = -1.5 (/24 hrs) = 24 -1.5 (/24 hrs) = 22.5 (/24 hrs)’.

### Appendix C

Provided [WBC] changes in the following manner: ‘5000 (/mm^3^) (= W_1_) →18000 (/mm^3^) (=W_2_). Because ‘W_1_ # W_(1→2)_ = W_2_’, the operator denoted by ‘W_(1→2)_’ for addition is derived from ‘W_(1→2)_ = W_2_ - W_1_ = 18000 - 5000 = 13000 (/mm^3^)’. Collectively, the operator is determined by division: ‘W_(1→2)_ = W_2_/W_1_ =18000/5000 (= 3.6) (/mm^3^)’,

For an another example, if ‘[Na]_1_ = 145 (mEq/l)’ changes into ‘[Na]_2_ = 128 (mEq/l)’, because ‘[Na]_1_ # [Na]_(1→2)_ = [Na]_2_’, the operator for addition is obtain from ‘[Na]_(1→2)_ = [Na]_2_ - [Na]_1_ = 128 - 145 = - 17 (mEq/l)’. Collectively, the operator for division is ‘[Na]_(1→2)_ = [Na]_2_/[Na]_1_ = 128/145 (mEq/l)’.

### Appendix D

R_(1→2)_ = R_2_ - R_1_

= [5 (mod 7) | 19.5 (/24 hrs) | 18000 (/mm^3^) | 128 (mEq/l) | X_(2)5_ (…)] - [2 (mod 7) | 21 (/24 hrs) | 5000 (/mm^3^) | 145 (mEq/l) | X_(1)5_ (…)],

= [5 - 2 (mod 7) | 19.5 - 21 (/24 hrs) | 18000 - 5000 (/mm^3^) | 128 - 145 (mEq/l) | X_(1→2)5_ (…)],

= [3 (mod 7) | - 1.5 (/24 hrs) | 13000 (/mm^3^) | - 17 (mEq/l) | X_(1→2)5_ (…)].

R_(2→3)_ = R_3_ - R_2_

= [3 (mod 7) | 22 (/24 hrs) | 7000 (/mm^3^) | 158 (mEq/l)] | X_(3)5_ (…)] - [5 (mod 7) | 19.5 (/24 hrs) | 18000 (/mm^3^) | 128 (mEq/l) | X_(2)5_ (…)],

= [3 - 5 (mod 7) | 22 - 19.5 (/24 hrs) | 7000 - 18000 (/mm^3^) | 158 - 128 (mEq/l) | X_(2→3)5_ (…)],

= [- 2 (mod 7) | 2.5 (/24 hrs) | - 11000 (/mm^3^) | 30 (mEq/l) | X_(2→3)5_ (…)],

= [5 (mod 7) | 2.5 (/24 hrs) | - 11000 (/mm^3^) | 30 (mEq/l) | X_(2→3)5_ (…)].

### Appendix E

R_1_◊R_(1→2)_◊R_(2→3)_ = [2 (mod 7) | 21 (/24 hrs) | 5000 (/mm^3^) | 145 (mEq/l) | X_(1)5_ (…)]◊[3 (mod 7) | - 1.5 (/24 hrs) | 13000 (/mm^3^) | - 17 (mEq/l) | X_(1→2)5_ (…)]◊[5 (mod 7) | 2.5 (/24 hrs) | - 11000 (/mm^3^) | 30 (mEq/l) | X_(2→3)5_ (…)],

= [2 + 3 + 5 (mod 7) | 21 - 1.5 + 2.5 (/24 hrs) | 5000 + 13000 - 11000 (/mm^3^) | 145 - 17 +30 (mEq/l) | X_(3)5_ (…)],

= [10 (mod 7) | 22 (/24 hrs) | 7000 (/mm^3^) | 158 (mEq/l) | X_(3)5_ (…)],

= [3 (mod 7) | 22 (/24 hrs) | 7000 (/mm^3^) | 158 (mEq/l) | X_(3)5_ (…)].

### Appendix F

For the 3rd and 4th components, only addition/subtraction is demonstrated collectively for ease in comprehension.

R_(2→10)_ = R_10_ - R_2_ = [0 - 5 (mod 7) | 17 - 19.5 (/24 hrs) | 9000 - 18000 (/mm^3^) | 130 - 128 (mEq/l) | X_(2→10)5_ (…)] = [- 5 (mod 7) | - 2.5 (/24 hrs) | - 9000 (/mm^3^) | 2 (mEq/l) | X_(2→10)5_ (…)],

R_(10→11)_ = R_11_ - R_10_ = [6 - 0 (mod 7) | 20 - 17 (/24 hrs) | 20000 - 9000 (/mm^3^) | 149 - 130 (mEq/l) | X_(10→11)5_ (…)] = [6 (mod 7) | 3 (/24 hrs) | 11000 (/mm^3^) | 19 (mEq/l) | X_(10→11)5_ (…)],

R_(11→12)_ = R_12_ - R_11_ = [4 - 6 (mod 7) | 23 - 20 (/24 hrs) | 6000 - 20000 (/mm^3^) | 140 - 149 (mEq/l) | X_(11→12)5_ (…)] = [- 2 (mod 7) | 3 (/24 hrs) | - 14000 (/mm^3^) | - 9 (mEq/l) | X_(11→12)5_ (…)],

R_(10→13)_ = R_13_ - R_10_ = [1 - 0 (mod 7) | 18 - 17 (/24 hrs) | 5000 - 9000 (/mm^3^) | 135 - 130 (mEq/l) | X_(10→13)5_ (…)] = [1 (mod 7) | 1 (/24 hrs) | - 4000 (/mm^3^) | 5 (mEq/l) | X_(10→13)5_ (…)],

R_(13→17)_ = R_17_ - R_13_ = [2 - 1 (mod 7) | 23.5 - 18 (/24 hrs) | 3000 - 5000 (/mm^3^) | 150 - 135 (mEq/l) | X_(13→17)5_ (…)] = [1 (mod 7) | 5.5 (/24 hrs) | - 2000 (/mm^3^) | 15 (mEq/l) | X_(13→17)5_ (…)].

## Competing interests

The authors declare that they have no competing interests.

## Authors’ contributions

JS conceived the main concept of this article and wrote the manuscript. SM revised the manuscript. JI gave advice on the potential validity from the viewpoint of clinical research and treatment. Additionally, all authors read and approved the final manuscript.
